# N-acetylcysteine inhibits *in vivo* oxidation of native low-density lipoprotein

**DOI:** 10.1038/srep16339

**Published:** 2015-11-05

**Authors:** Yuqi Cui, Chandrakala A. Narasimhulu, Lingjuan Liu, Qingbin Zhang, Patrick Z. Liu, Xin Li, Yuan Xiao, Jia Zhang, Hong Hao, Xiaoyun Xie, Guanglong He, Lianqun Cui, Sampath Parthasarathy, Zhenguo Liu

**Affiliations:** 1 Department of Cardiology, Shandong Provincial Hospital affiliated to Shandong University, Jinan, Shandong, China; 2 Dorothy M. Davis Heart and Lung Research Institute, Division of Cardiovascular Medicine, Department of Internal Medicine, Wexner Medical Center, The Ohio State University, Columbus, OH, USA; 3 Burnett School of Biomedical Sciences, University of Central Florida College of Medicine, USA

## Abstract

Low-density lipoprotein (LDL) is non-atherogenic, while oxidized LDL (ox-LDL) is critical to atherosclerosis. N-acetylcysteine (NAC) has anti-atherosclerotic effect with largely unknown mechanisms. The present study aimed to determine if NAC could attenuate *in vivo* LDL oxidation and inhibit atherosclerosis. A single dose of human native LDL was injected intravenously into male C57BL/6 mice with and without NAC treatment. Serum human ox-LDL was detected 30 min after injection, reached the peak in 3 hours, and became undetectable in 12 hours. NAC treatment significantly reduced serum ox-LDL level without detectable serum ox-LDL 6 hours after LDL injection. No difference in ox-LDL clearance was observed in NAC-treated animals. NAC treatment also significantly decreased serum ox-LDL level in patients with coronary artery diseases and hyperlipidemia without effect on LDL level. Intracellular and extracellular reactive oxidative species (ROS) production was significantly increased in the animals treated with native LDL, or ox-LDL and in hyperlipidemic LDL receptor knockout (LDLR^−/−^) mice that was effectively prevented with NAC treatment. NAC also significantly reduced atherosclerotic plaque formation in hyperlipidemic LDLR^−/−^ mice. NAC attenuated *in vivo* oxidation of native LDL and ROS formation from ox-LDL associated with decreased atherosclerotic plaque formation in hyperlipidemia.

Atherosclerosis is the most common cause of cardiovascular diseases like coronary artery disease (CAD) and stroke^1^. Hyperlipidemia is a major risk factor for the development of atherosclerosis. Although elevated low-density lipoprotein (LDL) is closely related to atherosclerosis, LDL itself is not atherogenic^2^,^3^. It is accepted that LDL oxidative modification with formation of oxidized LDL (ox-LDL) renders LDL atherogenic^4^,^5^,^6^. Indeed, after injection of unmodified human LDL to Sprague-Dawley rats, ox-LDL was detected in arterial endothelium^7^, suggesting that native LDL was converted to ox-LDL *in vivo*.

Oxidative stress with reactive oxygen species (ROS) formation plays a critical role in atherosclerosis^8^,^9^,^10^,^11^,^12^,^13^. ROS generation in blood monocytes is increased in hyperlipidemic patients with elevated plasma ox-LDL^10^. Ox-LDL is a potent oxidative agent that produces a significant amount of ROS *in vitro*^8^, and increases intracellular ROS formation in cultured endothelial cells^8^,^14^,^15^. ROS formation from ox-LDL is partially responsible for the actions of ox-LDL on bone marrow stem cells^8^.

N-acetylcysteine (NAC) inhibits the progression of atherosclerosis in apolipoprotein E-deficient mice^16^, decreases ROS generation and suppresses foam cell formation in the presence of ox-LDL^17^. NAC inhibits *in vitro* LDL oxidation induced by copper sulfate, 2,2′-azobis(2-amidinopropane) dihydrochloride, and UV light^18^. However, it is not known if native LDL oxidation to ox-LDL *in vivo* could be inhibited by NAC. The present study was to test the hypothesis that NAC decreased native LDL *in vivo* oxidation to ox-LDL and attenuated the progression of atherosclerosis. To achieve the goal, human native LDL was injected into male C57 BL/6 mice intravenously to determine the formation of oxidized human LDL with and without NAC treatment. *In vivo* ROS formation was determined in the mice injected with native LDL or ox-LDL and hyperlipidemic mice with and without NAC treatment. Our data demonstrated that native LDL was indeed converted to ox-LDL *in vivo* and generated a significant level of ROS that was effectively inhibited by NAC. NAC treatment did not affect the fate of ox-LDL *in vivo*. However, NAC effectively prevented the *in vivo* ROS production from ox-LDL and significantly reduced the progression of atherosclerotic lesions in LDL receptor knock-out (LDLR^−/−^) hyperlipidemic mice. NAC treatment also significantly decreased serum ox-LDL level in CAD patients with hyperlipidemia.

## Materials and Methods

### Preparation of native LDL, ox-LDL, DiI-LDL, and saturated LDL

Following Institutional Review Board approval, native LDL was prepared from the plasma from consented adult healthy donors by sodium bromide stepwise density gradient centrifugation as described^19^. Ox-LDL was prepared by exposure of native LDL to copper sulphate (5 μM) at 37 °C for 3 hrs^20^. Human native LDL was labeled with fluorescent dye 3,3′-dioctadecylindocarbocyanine (**Dil-LDL**, Enzo Life Sciences International, PA, USA) as described^21^. To exclude non-specific LDL oxidation *in vivo* and binding for ox-LDL detection assay, “**saturated LDL (Sat-LDL)**” with all the possible sites modified to prevent oxidation was prepared as the control LDL (Supplemental Fig. 1). The goal was to reduce unsaturated fatty acids to more saturated profile, thus minimizing oxidizability. See Supplemental Materials.

### Dynamics of native LDL and ox-LDL *in vivo*

All the animal experiments were performed in accordance with the “Guide for the Care and Use of Laboratory Animals of the US National Institutes of Health”. The experimental protocols were reviewed and approved by the Institutional Animal Care and Use Committee of the Ohio State University Wexner Medical Center, Columbus, Ohio, USA. After a single bolus injection of Dil-LDL or ox-LDL (50 μg) via tail vein, the serum, liver and spleen were obtained from the mice at different times to determine the level of Dil-LDL by detecting the fluorescence intensity as described^21^. PBS served as baseline control. Serum ox-LDL level at different time after injection was determined using human ox-LDL ELISA kit (Mercodia Inc. Winston Salem, NC, USA) as described^22^. Human Sat-LDL served as the control. The LDL preparations were made from human donors due to the fact that there was no antibody available to detect mouse ox-LDL. To evaluate the effect of NAC on the fate of native LDL and ox-LDL *in vivo*, the animals were treated with NAC (1 mg/mL in the drinking water) as described^23^. See Supplemental Materials.

### *In vivo* oxidation of native LDL

To demonstrate ox-LDL formation from native LDL *in vivo*, a single bolus dose of human native LDL (50 μg) was injected into the mice (male C57 BL/6, 6–8 weeks old) via tail vein with human Sat-LDL as control. The serum ox-LDL level was determined at different time points after injection as described above. To determine if NAC could affect the *in vivo* oxidation of native LDL, the mice were pre-treated with NAC as described above.

### Intracellular and extracellular ROS detection

Blood was harvested from the mice after intravenous injection of native LDL or ox-LDL (50 μg a day for 3 days), and in the LDLR^−/−^ mice after 4 months of high-fat diet (HFD) with and without NAC treatment for quantitative intracellular and extracellular ROS formation analysis using ROS Detection Reagents-FITC and electron paramagnetic resonance (EPR) spectroscopy, respectively, as described^24^. See Supplemental Materials.

### Animal model and atherosclerotic plaque ratio calculation

LDLR^−/−^ male mice with C57BL/6 background were fed with HFD for 4 months to induce hyperlipidemia and atherosclerosis with age-matched LDLR^−/−^ mice and C57BL/6 mice with normal diet (ND) as control. To evaluate the effect of NAC, some animals were treated with NAC orally for 4 months as described^23^. To determine if there was a time-dependent effect of NAC on atherosclerotic lesions, some hyperlipidemic mice were treated with NAC for 2 months after 2 months of HFD diet. The animals were then sacrificed to determine blood lipid profile, intracellular and extracellular ROS production, and aortic atherosclerotic lesions. The aorta was dissected from the aortic valve to the aortic hiatus. The atherosclerotic plaque was stained with oil red, and was quantitatively analyzed against the total aortic inner surface area as described^25^.

### Patient selection and human ox-LDL measurement

The patient study was conducted at the Shangdong University School of Medicine affiliated hospital in Jinan, Shangdong Province, China. The protocol was reviewed and approved by the university ethical review board. All patients provided their written informed consent. A total of 10 patients who had CAD and hyperlipidemia with age of at least 21 years old were recruited into the study. Age- and sex-matched healthy volunteers were recruited as the control. Patients were randomly divided into 2 groups with 5 patients in each group: NAC treatment group and placebo control. Baseline fasting lipid profile, serum ox-LDL level, blood glucose, thyroid stimulating hormone (TSH), kidney and liver functions were obtained from all the patients. Patients in the NAC treatment group received 250 mg NAC twice a day orally for 7 days with no further NAC treatment afterwards, while the patients in the control group were given placebo. The patients and the treating physicians had no knowledge on what they received (double blind). After one week of treatment and one week after discontinuing NAC, blood was collected to determine the fasting lipid profile, serum ox-LDL level, blood glucose, TSH, kidney and liver functions. The patients’ lipid profile was determined using an ARCHITECT ci16200 Integrated System (Abbott, Illinois, US) and an electrochemiluminescent procedure (Cobas E601; Roche, Basel, Switzerland). Patients’ serum ox-LDL was measured using human ox-LDL ELISA kit (Blue Gene, Shanghai, China). See Supplemental Materials.

### Statistical Analysis

The data were presented as means ± standard deviation (SD), and statistically analyzed using unpaired Student t-test (two-sided) for two groups of data or one way ANOVA (analysis of variance) (PRISM Version 4.0; GraphPad Software, Inc., San Diego, CA) followed by post hoc conservative Tukey’s test for three or more groups of data to minimize type I error as appropriate. Normal distribution of data was tested using the Shapiro–Wilk *W*-test, and equal variance was tested using the *F*-test. When the null hypothesis of normality and/or equal variance was rejected, the non-parametric Mann–Whitney *U*-test was used. When a two-tailed p < 0.05, the differences were considered statistically significant.

## Result

### *In vivo* kinetics of human native LDL and ox-LDL were very different in mice

**Figure 1:**
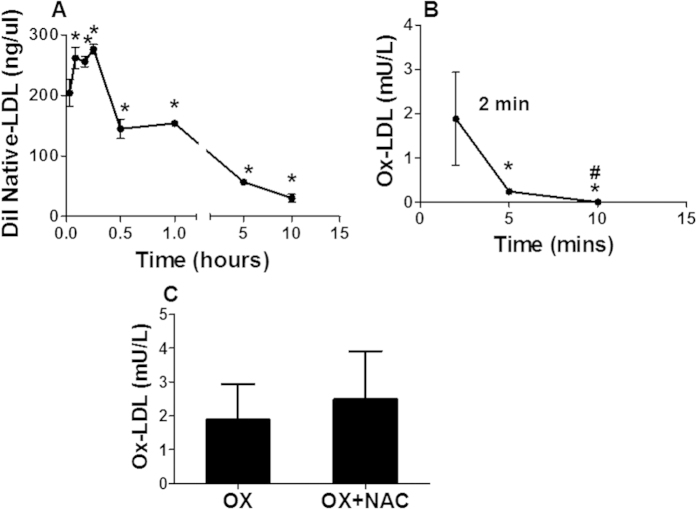
Dynamics of human native LDL and ox-LDL in the serum. Concentrations of human native LDL (**A**) and ox-LDL (**B**) in serum were determined in C57BL/6 mouse at different time points after a single injection of Dil human native LDL or ox-LDL. The native LDL level reached the peak level in the serum in 5 min and stayed at a significantly elevated level for 1 hour, then gradually decreased to undetectable level in 10 hours. On the other hand, the serum human ox-LDL level reached its peak in 2 min after injection, and became undetectable in 10 min (**B**). Treatment with NAC had no significant difference in the peak serum concentration of ox-LDL after intravenous administration in the mice (**C**). *p < 0.05, compared with 2 min, n = 5; ^#^p < 0.01, compared with 5 min, n = 5.

After a single intravenous injection of human Dil LDL in mice, the serum native LDL was rapidly increased in 2 min, reached the peak at 5 min, and remained stable until 15 min, then gradually declined to undetectable level by 10 hours after injection (Fig. 1A). However, the serum ox-LDL concentration reached the maximum within 2 min of injection, and rapidly declined to undetectable level within 10 min (Fig. 1B).

### Human native LDL was rapidly oxidized to ox-LDL and generated ROS *in vivo*

**Figure 2:**
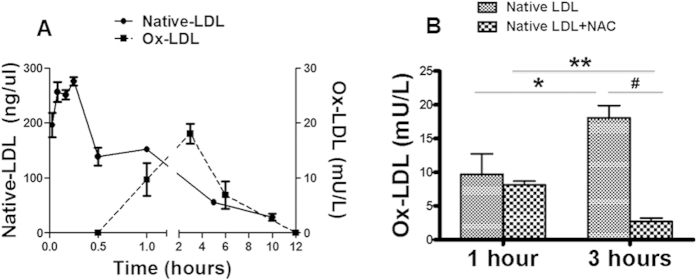
Native LDL conversion to ox-LDL *in vivo*. After a single injection of human native LDL to C57BL/6 mouse, the serum levels of native LDL and ox-LDL were determined at different time points. The native LDL level reached the peak in 5 min and stayed at a significantly elevated level for 1 hour, then gradually decreased to undetectable level in 10 hours. A measurable level of ox-LDL was detected 30 min after intravenous administration of native LDL. The serum ox-LDL level reached the peak in 3 hours, then started to decline, but stayed at detectable level until shortly after the disappearance of native LDL (**A**). The peak serum ox-LDL level at 3 hours after native LDL injection was dramatically decreased in the animals with NAC treatment (**B**). *P < 0.01, n = 5; **P < 0.001, ^#^P < 0.001, n = 5.

**Figure 3:**
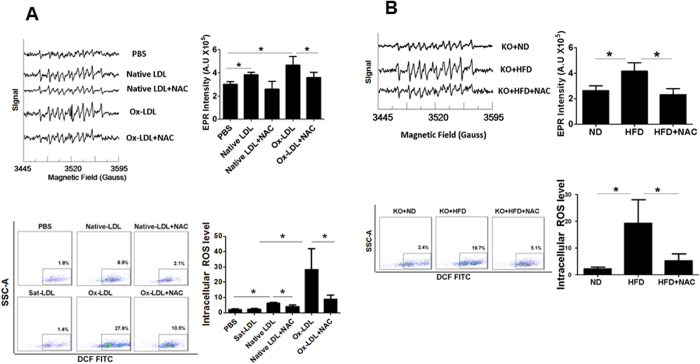
Extracellular and intracellular ROS formation. Extracellular ROS formation in the serum was quantitatively determined using EPR in the C57BL/6 mice after intravenous injection of human native LDL or human ox-LDL (once daily for three days) and in the hyperlipidemic LDLR^−/−^ mice with HFD for 4 months. Serum ROS production was significantly increased in the mice with native LDL or ox-LDL injection ((**A), upper panel**), and in the hyperlipidemic LDLR^−/−^ mice ((**B), upper panel**). Similarly, intracellular ROS production was quantitatively determined using the ROS Detection Reagents-FITC in the blood mononuclear cells. Intracellular ROS formation was significantly increased in the C57BL/6 mice with native LDL or ox-LDL injection ((**A), lower panel**) and the hyperlipidemic LDLR^−/−^ mice ((**B), lower panel**). Increased ROS production (both extracellular and intracellular) was completely blocked by NAC treatment in the mice received native LDL or ox-LDL, and in the hyperlipidemic LDLR^−/−^ mice (**A,B**). No significant change in ROS production was observed in the animals treated with Sat-LDL (control, (**A**)). **PBS:** C57BL/6 mouse with PBS injection; **Native LDL**: C57BL/6 mouse with human native LDL injection; **Native LDL+NAC**: C57BL/6 mouse with NAC treatment and human native LDL injection; **ox-LDL**: C57BL/6 mouse with human ox-LDL injection; **ox-LDL+NAC**: C57BL/6 mouse with NAC treatment and human ox-LDL injection; **Sat-LDL**: C57BL/6 mouse with Sat-LDL injection; **KO**+**ND**: LDLR^−/−^ mice with normal diet for 4 months; **KO+HFD**: LDLR^−/−^ mice with high fat diet for 4 months; **KO+HFD+NAC**: LDLR^−/−^ mice with high fat diet and NAC for 4 months. **SSC-A**: side scatter analysis. *P < 0.05, n = 5.

Native LDL was rapidly converted to ox-LDL with significant increase in serum ox-LDL by 30 min after injection. The maximal ox-LDL level was observed by 3 hours, then started to decline gradually afterwards with no detectable serum ox-LDL 12 hours after native LDL administration (Fig. 2A). A detectable level of both intracellular and extracellular ROS was generated in the animals after intravenous native LDL administration. Similar results on intracellular and extracellular ROS production were observed in the mice treated with ox-LDL injection. No *in vivo* ROS production was detected in the animals received Sat-LDL (Fig. 3A).

### NAC treatment prevented *in vivo* ROS production from ox-LDL without affecting ox-LDL kinetics

NAC could effectively block ROS formation from ox-LDL *in vitro*^8^. In the present study, we observed that NAC treatment almost completely prevented both intracellular and extracellular ROS formation from ox-LDL (Fig. 3A). Interestingly, the serum ox-LDL level was not significantly affected in the animals treated with NAC (Fig. 1C), suggesting that inhibition of *in vivo* ROS production from ox-LDL by NAC was not due to reduced serum ox-LDL.

### NAC attenuated *in vivo* oxidation of native LDL

A detectable ox-LDL level was present in the serum within 30 min after human native LDL injection, and reached the peak level in 3 hours (Fig. 2A). It was substantially reduced in the mice treated with NAC as compared with the control (Fig. 2B). *In vivo* intracellular and extracellular ROS production after native LDL administration was also significantly decreased in NAC-treated animals (Fig. 3A).

### NAC inhibited *in vivo* ROS generation and atherosclerotic plaque formation in hyperlipidemic mice

**Figure 4:**
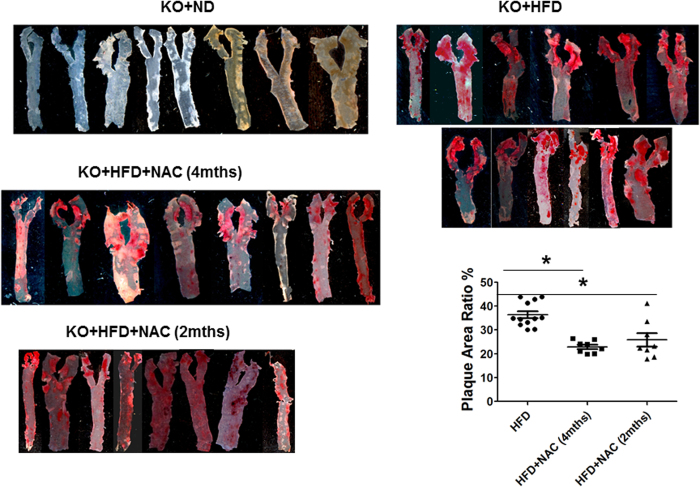
Mouse aortic atherosclerosis formation. The aorta was dissected from the aortic valve to the aortic hiatus and stained for atherosclerotic plaque analysis in the hyperlipidemic LDLR^−/−^ mice after 4 months of HFD diet. A significant amount of atherosclerotic plaque was identified in the hyperlipidemic LDLR^−/−^ mice, while no lesion was present in the age-matched wild-type control mice (**left panel**). NAC treatment for 2 or 4 months significantly reduced the level of atherosclerotic lesions in the hyperlipidemic LDLR^−/−^ mice (**right and left panels**). There was no statistical significance in the size of atherosclerotic plaque in the mice treated with NAC for 4 months and 2 months. **KO+HFD+NAC (4 mths)**: LDLR^−/−^ mice with high fat diet and NAC for 4 months. *P < 0.05, n = 8; **KO+HFD+NAC (2 mths)**: Ldlr^−/−^ mice with high fat diet for 4 months and started feeding NAC for 2 months after 2-month-HFD treatment. *P < 0.05, n = 8.

Total LDL and non-HDL lipoprotein levels were dramatically increased in LDLR^−/−^ mice with HFD (Supplemental Table 1), confirming a successful hyperlipidemic mouse model. Both intracellular and extracellular ROS production was significantly increased in hyperlipidemic mice over the control LDLR^−/−^ mice on ND (Fig. 3B). As expected, a significant amount of atherosclerotic plaque was present in the aorta in hyperlipidemic mice (Fig. 4). NAC treatment did not significantly change the lipid profile in hyperlipidemic mice (Supplemental Table 1). However, ROS production in the serum and in the mononuclear cells was significantly reduced in NAC-treated hyperlipidemic mice (Fig. 3B). The level of atherosclerotic plaque was also significantly decreased in the hyperlipidemic mice treated with NAC (Fig. 4). There was no statistically significant difference in the size of atherosclerotic plaque in the mice treated with NAC for 4 months and 2 months although treatment for 4 months seemed to reduce the atherosclerotic plaque further.

### NAC treatment selectively decreased serum ox-LDL level in hyperlipidemic patients

**Figure 5:**
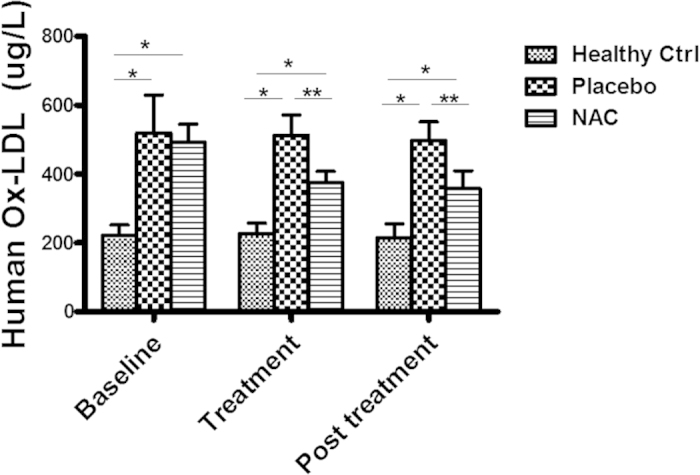
NAC treatment decreased serum ox-LDL Level in hyperlipidemic patients. Serum ox-LDL levels were measured using ELISA in the patients with documented coronary artery disease (CAD) and hyperlipidemia after one week of NAC treatment. The serum ox-LDL level was significantly elevated in the patients with hyperlipidemia as compared with the healthy volunteers. NAC treatment significantly decreased the serum ox-LDL level in the patients. The serum ox-LDL remained decreased in the patients one week after discontinuation of NAC. *P < 0.001, NAC treatment vs placebo (n = 5); **p < 0.01, n = 5.

Serum LDL, non-HDL lipoprotein level, and ox-LDL were all significantly increased in the patients with hyperlipidemia over the age- and sex-matched healthy individuals as expected (Supplemental Table 2). There were no significant differences in lipid profile and serum ox-LDL level in the patients of the two groups at baseline. After one week of NAC treatment, there were no significant changes in serum LDL, non-HDL lipoprotein levels, blood glucose, TSH, kidney and liver functions in the patients. However, the serum ox-LDL was significantly decreased in the patients with NAC treatment as compared with the placebo group (Fig. 5). Of note, decreased level of serum ox-LDL was still present in the patients one week after NAC discontinuation. Moreover, decreased level of serum ox-LDL was still present in the patients one week after NAC discontinuation. These data demonstrated that NAC treatment selectively decreased serum ox-LDL level, indicating that NAC might decrease *in vivo* oxidation of LDL in the patients.

## Discussion

It is believed that LDL *in vivo* oxidation into ox-LDL is a critical step for *in vivo* ROS production and atherosclerotic plaque formation in hyperlipidemia^4^,^5^,^6^. In the present study, we demonstrated that native LDL was indeed oxidized to ox-LDL *in vivo* with production of intracellular and extracellular ROS in mice. We also showed that intravenous ox-LDL administration generated a significant amount of ROS *in vivo*. Since native LDL *per se* did not generate ROS^8^, the *in vivo* ROS production after native LDL administration could be only from the ox-LDL generated *in vivo* from the native LDL. The data from the present study provided direct evidence that native LDL was oxidized *in vivo* with generation of ox-LDL and ROS.

The mechanism(s) for native LDL *in vivo* conversion to ox-LDL is very complex and remain largely unknown. Early studies indicated that LDL oxidative modification mainly occurred in macrophages and/or smooth muscle cells during the process of “foam cell” formation^26^,^27^. This theory was developed originally based on an *in vitro* observation that cell-modified (oxidized) LDL had the potential to promote foam cell formation when mixed with macrophages^28^,^29^. Native LDL oxidation could be induced by incubating LDL with the cells from artery, redox-active transition metals like iron or lipoxygenases, leading to physicochemical and biological changes (particularly atherogenic changes) of LDL molecules with the formation of ox-LDL^30^. *In vivo* studies indicated that ox-LDL and oxidized-like lipoproteins were present in the atheroma gruel, but not in the normal areas of aorta^31^,^32^,^33^,^34^. Ox-LDL was identified in the arterial endothelium and media within 6 hours after injection of unmodified human LDL in rats^7^. Therefore, LDL oxidation was considered to occur in the arterial intima. However, native LDL oxidation could occur in circulation based on an *in vitro* experiment that lipolysis-induced acidic conditions enhanced iron release from transferrin and increased ox-LDL formation^35^. The data from the present study demonstrated that after injection of human native LDL into mouse, human ox-LDL was rapidly detected in blood. The appearance of serum ox-LDL seemed to be closely related to native LDL disappearance. Since ox-LDL could only stay in the circulation for a very short period of time (<10 min), which was consistent with previous observation^36^, ox-LDL generated from the native LDL could disappear from the circulation quickly when native LDL was no longer present. These data supported the rationale that LDL oxidation could occur in the blood instead of inside other tissues or organ systems since the levels of native LDL in the liver and spleen were dramatically different (it remained at high level for over 10 hours in the liver, and disappeared within 15 min in the spleen) (Supplemental Fig. 2). However, further studies are needed to investigate the location(s) of LDL oxidation and related mechanism(s) *in vivo*.

A variety of antioxidants have been tested to evaluate their potential effects on hyperlipidemia and atherosclerosis in many different animal models. Despite strong pre-clinical evidence and promising data in animal studies, most clinical trials on antioxidants including vitamin E or β-carotene have failed to achieve significant clinical benefits (if not harmful) in patients with cardiovascular diseases^2^,^3^,^37^. NAC has been traditionally considered as an antioxidant and decreases the progression of atherosclerotic lesions in animal studies^16^,^38^. The present study demonstrated that NAC effectively inhibited native LDL *in vivo* oxidation and ROS formation in association with decreased atherosclerosis. NAC treatment also significantly and selectively decreased serum ox-LDL level in hyperlipidemic patients. Since NAC did not change serum LDL level nor affect the *in vivo* dynamics of ox-LDL, the decreased serum ox-LDL level in hyperlipidemic patients by NAC could be only due to inhibition of *in vivo* LDL oxidation. Lipoprotein(a) [Lp(a)] is a complex of LDL linked by disulphide bridges with apo(a), and is associated with atherosclerotic disease. The effect of NAC on plasma Lp(a) has not been well defined. Some studies showed that NAC had no effect on plasma Lp(a)^39^, while a small but significant decrease in Lp(a) concentration (7%) was observed after NAC treatment in patients^40^. In the present study, NAC treatment had no effect on plasma LDL concentration in hyperlipidemic patients or mice. However, NAC treatment significantly decreased serum ox-LDL level in hyperlipidemic patients. These observations clearly raise the possibility that NAC is more than an ordinary antioxidant and its different effect on the lipid profiles remains unknown. Clinical studies are needed to confirm the beneficial effect of NAC on the progression of atherosclerotic lesions in CAD patients. Further studies are also needed to establish the direct relationship between attenuation of *in vivo* LDL oxidation and reductions of atherosclerosis in hyperlipidemic animal models like LDL receptor knockout mice.

Ox-LDL is important to atherosclerosis at all stages from inducing endothelial cell apoptosis and foam cell formation to fibrous cap rupture and acute thrombotic events through various mechanisms^41^,^42^. An important mechanism for the actions of ox-LDL is ROS formation and oxidative stress^7^,^9^,^10^. Indeed, a significant amount of ROS was released spontaneously from ox-LDL, not from native LDL, *in vitro*^8^. In the present study, we observed that ox-LDL, but not sat-LDL, enhanced both intracellular and extracellular ROS production *in vivo.* Increased intracellular and extracellular ROS formation *in vivo* was also observed in hyperlipidemic mice. These data suggested that increased *in vivo* ROS production in hyperlipidemic states was very likely from ox-LDL generated from native LDL.

Very little is known on the mechanism(s) for ROS production from ox-LDL. ROS formation from ox-LDL *in vitro* was completely prevented with NAC^8^. Both intracellular and extracellular ROS production in ox-LDL-treated mice and hyperlipidemic mice was completely blocked by NAC treatment. The atherosclerotic plaque was also significantly decreased in NAC-treated hyperlipidemic mice, suggesting that NAC reduced atherosclerosis at least partially due to decreased *in vivo* ROS production (Supplemental Fig. 3). Of note, NAC almost completely blocked intracellular and extracellular ROS production in circulation in hyperlipidemic mice, while it only partially prevented formation of atherosclerotic lesions, indicating that ROS in the circulating monocytes and plasma was only partially involved in the development of atherosclerosis. Further studies are needed to investigate the role of ROS production in the aortic wall and other mechanism(s) in the formation of atherosclerotic lesions.

## Conclusions

These data suggested that NAC attenuated *in vivo* oxidation of native LDL and ROS formation from ox-LDL that might partially contribute to decreased atherosclerotic plaque formation in hyperlipidemia. Further studies are needed to investigate the role of ROS production in the aortic wall and other mechanism(s) in the formation of atherosclerotic lesions.

## Additional Information

**How to cite this article**: Cui, Y. *et al.* N-acetylcysteine inhibits *in vivo* oxidation of native low-density lipoprotein. *Sci. Rep.* **5**, 16339; doi: 10.1038/srep16339 (2015).

## Supplementary Material

Supplementary Dataset
