# Supplementary material for: N-acetylcysteine inhibits in vivo oxidation of native low-density lipoprotein
Source: Sci Rep. 2015 Nov 5;5:16339. doi: 10.1038/srep16339 (PMC4633729; doi:10.1038/srep16339)

**N-acetylcysteine inhibits *in vivo* oxidation of native low-density lipoprotein**

Yuqi Cui, MD, PhD^1,2^, Chandrakala A Narasimhulu, PhD^3^, Lingjuan Liu, MSc^2^, Qingbin Zhang, MD^1^, Patrick Z Liu^2^, Xin Li, MD^2^, Yuan Xiao, MD^2^, Jia Zhang, MD^2^, Hong Hao, PhD^2^, Xiaoyun Xie, MD, PhD^2^, Guanglong He, PhD^2^, Lianqun Cui, MD^1^, Sampath Parthasarathy, PhD^3^, Zhenguo Liu^2^*, MD, PhD

^1^ Department of Cardiology, Shandong Provincial Hospital, Shandong University, Jinan, Shandong, China; ^2^Dorothy M. Davis Heart and Lung Research Institute, Division of Cardiovascular Medicine, Department of Internal Medicine, Wexner Medical Center, The Ohio State University, Columbus, OH, USA; ^3^Burnett School of Biomedical Sciences, University of Central Florida College of Medicine, USA.

***Correspondence:** Zhenguo Liu, MD, PhD

Davis Heart & Lung Research Institute

The Ohio State University Wexner Medical Center

Room 200 DHLRI; 473 West 12^th^ Ave

Columbus, OH 43210, USA

Phone: (614)247-8435; Fax: 614-293-5614

E-mail: [zhenguo.liu@osumc.edu](mailto:zhenguo.liu@osumc.edu)

**Methods and Materials**

**Preparation of native LDL, ox-LDL, DiI-LDL, and saturated LDL**

Following Institutional Review Board approval, blood was collected in heparinized tubes from consented adult healthy donors, and stored on ice. Plasma was obtained from the blood after centrifuging at 1500g for 20 min. Lipoproteins were isolated from the plasma using sequential ultracentrifugation with a Beckman TL-100 tabletop ultracentrifuge (Beckman, Palo Alto, CA) as described [^1^](#_ENREF_1). The isolated lipoproteins were dialyzed against 0.3 mM EDTA in 1x phosphate buffer saline (PBS, pH 7.4) overnight and subsequently sterilized with 0.22 μM filter. The protein concentration in the lipoproteins was determined using the Folin Lowry method. Ox-LDL was produced from the native LDL immediately after dialysis using 5 μM copper sulphate and was stopped by adding EDTA (0.25 mM, final concentration) as described [^2^](#_ENREF_2). Human native LDL was labeled with fluorescent dye DiI (3,3'-dioctadecylindocarbocyanine, Cat:ENZ-52206, Enzo Life Sciences International, INC. PA, USA) as described previously [^3^](#_ENREF_3). Briefly, 1 mg of LDL was incubated with 2 ml of lipoprotein-deficient serum and 50 μl of DiI in dimethyl sulfoxide (3 mg/ml stock) at 37°C for 15h. It was then stored at 4°C, and used within 1 week of preparation.

To exclude non-specific LDL oxidation *in vivo* and binding for the detection assay for ox-LDL, "**saturated LDL (Sat-LDL)**" with all the possible sites modified to prevent oxidation was prepared as the control LDL. The goal was to reduce unsaturated fatty acids to more saturated profile, thus minimizing oxidizability. A total of 2 mg native LDL was placed in a glass tube, and the volume was adjusted to 500 μl with PBS. A total of 2 mg sodium borohydride was dissolved in 100 μl of PBS, and mixed with LDL, kept on ice, over a period of 2 hours at 5 minutes interval. The sample was mildly stirred during the process of preparation. After the final addition, the sample was dialyzed and filtered sterile. The electrophoretic mobility, protein, and cholesterol levels were determined to ensure sample purity. Lack of conjugated diene formation upon oxidation with copper was used for the evaluation of saturation. Overall, the Sat-LDL exhibited the same electrophoretic mobility as native LDL (**Supplemental Fig. 1**), had 96% of the cholesterol/protein as the original LDL, and was very resistant to oxidation. Less than 20% oxidation was noted after copper oxidation. The Sat-LDL was used as control.

**Dynamics of native LDL and ox-LDL *in vivo***

All the animal experiments were performed in accordance with the “Guide for the Care and Use of Laboratory Animals of the US National Institutes of Health”. The experimental protocols for the present study were reviewed and approved by the Institutional Animal Care and Use Committee of the Ohio State University Wexner Medical Center, Columbus, Ohio, USA. After a single bolus injection of human Dil-LDL or ox-LDL (50 μg) via tail vein, the mice (male C57 BL/6, 6-8 weeks old, from Jackson Laboratory, ME, USA) were sacrificed to obtain the serum, liver and spleen at different times to determine the level of Dil-LDL by detecting the fluorescence intensity in the preparations (excitation 549nm, emission 565nm) as described [^3^](#_ENREF_3). A total of 160 μl serum was used to determine the blood Dil-LDL level for each mouse. A total of 100 mg of liver and 20 mg of spleen were homogenized using PBS in a total volume of 170 μl to determine their Dil-LDL levels. PBS was used as baseline control. To measure the serum level of human ox-LDL, blood was collected at different time points (at least 5 mice for each time point), and centrifuged at 1600g for 15 min at 4 °C degree to obtain the serum as described [^4^](#_ENREF_4). Human Sat-LDL was used as the control. Serum ox-LDL level at different time after injection was determined using human ox-LDL ELISA kit (Mercodia Inc. Cat: 10-1143-01, Winston Salem, NC, USA) as described [^5^](#_ENREF_5). To evaluate the effect of NAC on the fate of native LDL and ox-LDL *in vivo*, the animals were treated with NAC (Sigma, Cat: A7250, 1mg/mL in the drinking water) as described [^6^](#_ENREF_6).

***In vivo* oxidation of native LDL**

To demonstrate ox-LDL formation from native LDL *in vivo*, a single bolus dose of human native LDL (50 μg) was injected into the mice (male C57 BL/6, 6-8 weeks old) via tail vein with human Sat-LDL as control. The serum ox-LDL level was determined at different time points after injection as described above. To determine if NAC could affect the *in vivo* oxidation of native LDL, the mice were pre-treated with NAC as described above.

**Intracellular and extracellular ROS detection**

Blood was harvested from the mice after injection of native LDL or ox-LDL (50 μg) for 3 days via tail vein, and in the hyperlipidemic LDL receptor knockout (LDLR^-/-^) mice after 4 months of high-fat diet with and without NAC treatment (as detailed below). Red blood cells were eliminated using red blood cell lysis as described [^7^](#_ENREF_7). The level of intracellular ROS formation in blood cells after injection of native LDL or ox-LDL was determined using the ROS Detection Reagents-FITC (Invitrogen, Cat: D399) as described [^8^](#_ENREF_8). The cells were incubated with the reagent for 10 min at 37°C. The labeled cells were washed twice with PBS and then suspended in warm PBS for analysis using flow cytometry. The fluorescence-positive cells were quantitatively evaluated using an LSRII (BD Bioscience, CA, USA) at the wavelength of 525nm as described [^9^](#_ENREF_9). Extracellular ROS formation in the serum was quantitatively determined using the *ex vivo* electron paramagnetic resonance (EPR) spectroscopy with 5-diethoxyphosphoryl-5-methyl-1-pyrroline N-oxide (DEPMPO) (Santa Cruz, Cat: sc-202132) as the spin trap as described. ROS production was measured using EPR spectroscopy through mixture of 50 mM DEPMPO (final concentration) with serum as described [^10^](#_ENREF_10).

**Animal model and atherosclerotic plaque ratio calculation**

LDLR^-/-^ male mice with C57BL/6 background (6-8 weeks old, from Jackson Laboratory, ME, US) were obtained from Jackson Laboratory (ME, US). When the mice reached the age of 8 weeks, the animals were fed with high fat diet (**HFD**) (17% AMF and 0.2% cholesterol, Harlan, Cat: TD04287, WI, USA) for 4 months to induce hyperlipidemia and atherosclerosis. Age-matched C57BL/6 wild type and LDLR^-/-^ male mice with normal diet were used as control. To evaluate the effect of NAC on blood lipid profile, *in vivo* ROS production, and atherosclerotic plaque formation in the hyperlipidemic mice, some animals were treated with NAC orally (1 mg/mL in drinking water, average 503.5 mg/kg/day) for 4 months as described [^6^](#_ENREF_6). After 4 months of HFD, the animals were sacrificed to collect the blood to determine blood lipid profile, intracellular and extracellular ROS production, and aorta for atherosclerotic plaque formation. The atherosclerotic plaque was stained with oil red, and the plaque area was quantitatively analyzed against the total inner surface area of the aorta as described [^11^](#_ENREF_11).

**Patient selection and human ox-LDL measurement**

The patient study was conducted at the Shangdong University School of Medicine affiliated hospital in Jinan, Shangdong Province, China. The protocol was reviewed and approved by the university ethical review board. All patients provided their written informed consent. A total of 10 patients who had CAD and hyperlipidemia with ago of at least 21 years old were recruited into the study. Age- and sex-matched healthy volunteers were recruited as the control. Patients were randomly divided into 2 groups with 5 patients in each group: NAC treatment group and placebo control. Baseline fasting lipid profile, serum ox-LDL level, blood glucose, thyroid stimulating hormone (TSH), kidney and liver functions were obtained for all the patients. Patients in the NAC treatment group received 250 mg NAC twice a day orally for 7 days with no further NAC treatment afterwards, while the patients in control group were given placebo. The patients and the treating physicians had no knowledge on what they received (double blind). After one week of treatment and one week after discontinuing NAC, blood was collected to determine the fasting lipid profile, serum ox-LDL level, blood glucose, TSH, kidney and liver functions. The patients’ lipid profile was determined using an ARCHITECT ci16200 Integrated System (Abbott, Illinois, US) and an electrochemiluminescent procedure (Cobas E601; Roche, Basel, Switzerland). Patients’ serum ox-LDL was measured using human ox-LDL ELISA kit (Blue Gene, Cat: E01O0026, Shanghai, China).

**Statistical Analysis**

The data were presented as means ± standard deviation (SD), and statistically analyzed using unpaired Student t-test (two-sided) for two groups of data or one way ANOVA (analysis of variance) (PRISM Version 4.0; GraphPad Software, Inc., San Diego, CA) followed by post hoc conservative Tukey’s test for three or more groups of data to minimize type I error as appropriate. Normal distribution of data was tested using the Shapiro–Wilk *W*-test and equal variance was tested using the *F*-test. When the null hypothesis of normality and/or equal variance was rejected, the non-parametric Mann–Whitney *U*-test was used. When a two-tailed p < 0.05, the differences were considered statistically significant.

**References**

1 Chung, B. H., Wilkinson, T., Geer, J. C. & Segrest, J. P. Preparative and quantitative isolation of plasma lipoproteins: rapid, single discontinuous density gradient ultracentrifugation in a vertical rotor. *J Lipid Res* **21**, 284-291 (1980).

2 Chandrakala, A. N. *et al.* Induction of brain natriuretic peptide and monocyte chemotactic protein-1 gene expression by oxidized low-density lipoprotein: relevance to ischemic heart failure. *American journal of physiology. Cell physiology* **302**, C165-177, doi:10.1152/ajpcell.00116.2011 (2012).

3 Pitas, R. E., Innerarity, T. L., Weinstein, J. N. & Mahley, R. W. Acetoacetylated Lipoproteins Used to Distinguish Fibroblasts from Macrophages Invitro by Fluorescence Microscopy. *Arteriosclerosis* **1**, 177-185 (1981).

4 Beckonert, O. *et al.* Metabolic profiling, metabolomic and metabonomic procedures for NMR spectroscopy of urine, plasma, serum and tissue extracts. *Nat Protoc* **2**, 2692-2703, doi:DOI 10.1038/nprot.2007.376 (2007).

5 Rao, V. S., Nagaraj, R. K., Hebbagodi, S., Kadarinarasimhiah, N. B. & Kakkar, V. V. Association of inflammatory and oxidative stress markers with metabolic syndrome in asian indians in India. *Cardiology research and practice* **2011**, 295976, doi:10.4061/2011/295976 (2010).

6 Jang, Y. Y. & Sharkis, S. J. A low level of reactive oxygen species selects for primitive hematopoietic stem cells that may reside in the low-oxygenic niche. *Blood* **110**, 3056-3063, doi:10.1182/blood-2007-05-087759 (2007).

7 Houlihan, D. D. *et al.* Isolation of mouse mesenchymal stem cells on the basis of expression of Sca-1 and PDGFR-alpha. *Nat Protoc* **7**, 2103-2111, doi:10.1038/nprot.2012.125 (2012).

8 Bilski, P., Belanger, A. G. & Chignell, C. F. Photosensitized oxidation of 2 ',7 '-dichlorofluorescin: Singlet oxygen does not contribute to the formation of fluorescent oxidation product 2 ',7 '-dichlorofluorescein. *Free Radical Bio Med* **33**, 938-946, doi:Pii S0891-5849(02)00982-6 Doi 10.1016/S0891-5849(02)00982-6 (2002).

9 Robinson, J. P. *et al.* Measurement of Intracellular Fluorescence of Human-Monocytes Relative to Oxidative-Metabolism. *J Leukocyte Biol* **43**, 304-310 (1988).

10 Lu, T. W. *et al.* Reactive Oxygen Species Mediate Oxidized Low-Density Lipoprotein-Induced Inhibition of Oct-4 Expression and Endothelial Differentiation of Bone Marrow Stem Cells. *Antioxid Redox Sign* **13**, 1845-1856, doi:DOI 10.1089/ars.2010.3156 (2010).

11 Chang, P. C., Wu, H. L., Lin, H. C., Wang, K. C. & Shi, G. Y. Human plasminogen kringle 1-5 reduces atherosclerosis and neointima formation in mice by suppressing the inflammatory signaling pathway. *J Thromb Haemost* **8**, 194-201, doi:DOI 10.1111/j.1538-7836.2009.03671.x (2010).

**Supplemental t**able 1. Mouse serum lipid profile

|  | **WT+ND**  **(Mean±SD, n=8)** | **KO+ND**  **(Mean±SD, n=8)** | **KO+HFD**  **(Mean±SD, n=12)** | **KO+HFD+NAC (4mths) (Mean±SD,n=8)** | **KO+HFD+NAC (2mths) (Mean±SD,n=8)** |
| --- | --- | --- | --- | --- | --- |
| **TC**  **( mg/dl)** | **<100** | **270.2 ± 38.9** ***** | **1732.4 ± 424.1** ****** | **1428 ± 256.2** | **2060 ± 310.9** |
| **HDL**  **(mg/dl)** | **80.8 ± 3** | **82.2 ± 2.6** | **93.6 ± 17.3**** | **84.8 ± 18.2** | **97.5 ± 13.1** |
| **TRG**  **(mg/dl)** | **118 ± 7.3** | **167.7 ± 49.9 *** | **465.8 ± 215.8**** | **334.4 ± 109.2** | **365 ± 152** |
| **LDL**  **(mg/dl)** | **<100** | **145 ± 27.6 *** | **1349.8 ± 167.6**** | **1276.0 ± 265.4** | **1286.3 ± 283.8** |
| **Non-HDL**  **(mg/dl)** | **<100** | **181.8 ± 38.9 *** | **1440.6 ± 202.9**** | **1343.2 ± 273.7** | **1363.8 ± 309.6** |
| **TC/HDL** | **<2** | **3.3 ± 0.5 *** | **18.3 ± 5.4**** | **17.9 ± 6.8** | **21.5 ± 4** |

C57BL/6 and LDLR^-/-^ mice were fed with normal diet or high fat diet for 4 months as indicated with or without NAC for 4 months or 2 months after 2-month-HFD treatment. Serum lipid profile was determined as listed in the table. **WT**+**ND**: wild type mice with normal diet for 4 months; **KO**+**ND**: LDLR^-/-^ mice with normal diet for 4 months; **KO+HFD**: LDLR^-/-^ mice with high fat diet for 4 months; **KO+HFD+NAC**: LDLR^-/-^ mice with high fat diet and NAC for 4 months. **HDL**: high density lipoprotein; **LDL**: low density lipoprotein; **TRG**: triglycerides; **TC**: total cholesterol,* p<0.001 (KO+ND vs WT+ND); ** p<0.001 (KO+HFD vs KO+ND).

**Supplemental t**able 2. Human plasma lipid profile

|  |  | **HDL(mmol/L)** | **LDL(mmol/L)** | **TG(mmol/L)** | **TC(mmol/L)** |
| --- | --- | --- | --- | --- | --- |
| **Baseline** | **Without treatment (Mean ±SD, n=5)** | **1.05 ± 0.22** | **2.82 ± 0.53** | **1.60 ± 0.13** | **4.86 ± 0.90** |
|  | **Without treatment (Mean ±SD, n=5)** | **1 ± 0.08** | **2.35 ± 0.59** | **2.05 ± 1.01** | **4.30 ± 0.88** |
| **Treatment** | **Placebo**  **(Mean ±SD, n=5)** | **1.06 ± 0.16** | **2.84 ± 0.56** | **1.61 ± 0 ±12** | **4.83 ± 0.71** |
|  | **NAC**  **(Mean ±SD, n=5)** | **1.18 ± 0.15** | **2.67 ± 0.84** | **2.38 ± 0.94** | **4.73 ± 1.06** |
| **Post treatment** | **Placebo**  **(Mean ±SD, n=5)** | **1.05 ± 0.20** | **2.71 ± 0.60** | **1.47 ± 0.19** | **5.02 ± 0.60** |
|  | **NAC**  **(Mean ±SD, n=5)** | **1.11 ± 0.11** | **2.76 ± 0.73** | **1.91 ± 0.36 *** | **4.49 ± 0.71** |

Serum lipid profile was determined in the patients with coronary artery disease and hyperlipidemia with and without NAC treatment. **Baseline**: Patient without placebo or NAC treatment; **Treatment**: Patient with placebo or NAC treatment for 7 days; **Post treatment**: patient stopped treating placebo or NAC for 7 days. **HDL**: high density lipoprotein; **LDL**: low density lipoprotein; **TRG**: triglycerides; **TC**: total cholesterol. p<0.05 (NAC vs Placebo).

**Figure legends**

**Supplemental Figure 1. Electrophoretic mobility of native LDL and Sat-LDL.** To evaluate the *in vivo* oxidation of native LDL, saturated LDL (**Sat-LDL**) with all the possible sites modified to prevent oxidation was prepared as the control LDL to exclude non-specific LDL oxidation *in vivo* and reaction for the detection assay for ox-LDL. The electrophoretic mobility of native LDL and Sat-LDL was the same.

**Supplemental Figure 2. *In vivo* kinetics of human native LDL in the liver and spleen in mice**. Human native LDL concentrations in the liver and spleen were determined in 6-8 weeks old C57BL/6 mice at different time points after a single injection of Dil human native LDL. The native LDL level in the liver was low in the first 30 min after IV injection. However, it increased rapidly afterwards, reached the maximal level in the liver in 1 hour, and stayed significantly elevated thereafter for over 10 hours after a small decline one hour after administration (**A**). Interestingly, the native LDL level in spleen was the highest at 10 min after injection, and became undetectable within 15 min (**B**). n = 5.

**Supplemental Figure 3. Possible mechanisms on the beneficial effect of NAC on the reduction of atherosclerotic plaque in hyperlipidemia**. Native-LDL was increased in the hyperlipidemic state and was oxidized to ox-LDL *in vivo*. A significant amount of ROS was produced from ox-LDL *in vivo* which could contribute to the development of atherosclerosis. NAC attenuated the *in vivo* oxidation of native LDL with reduced formation of ox-LDL, and inhibited ROS production from ox-LDL, thus decreasing the amount of atherosclerotic lesions.


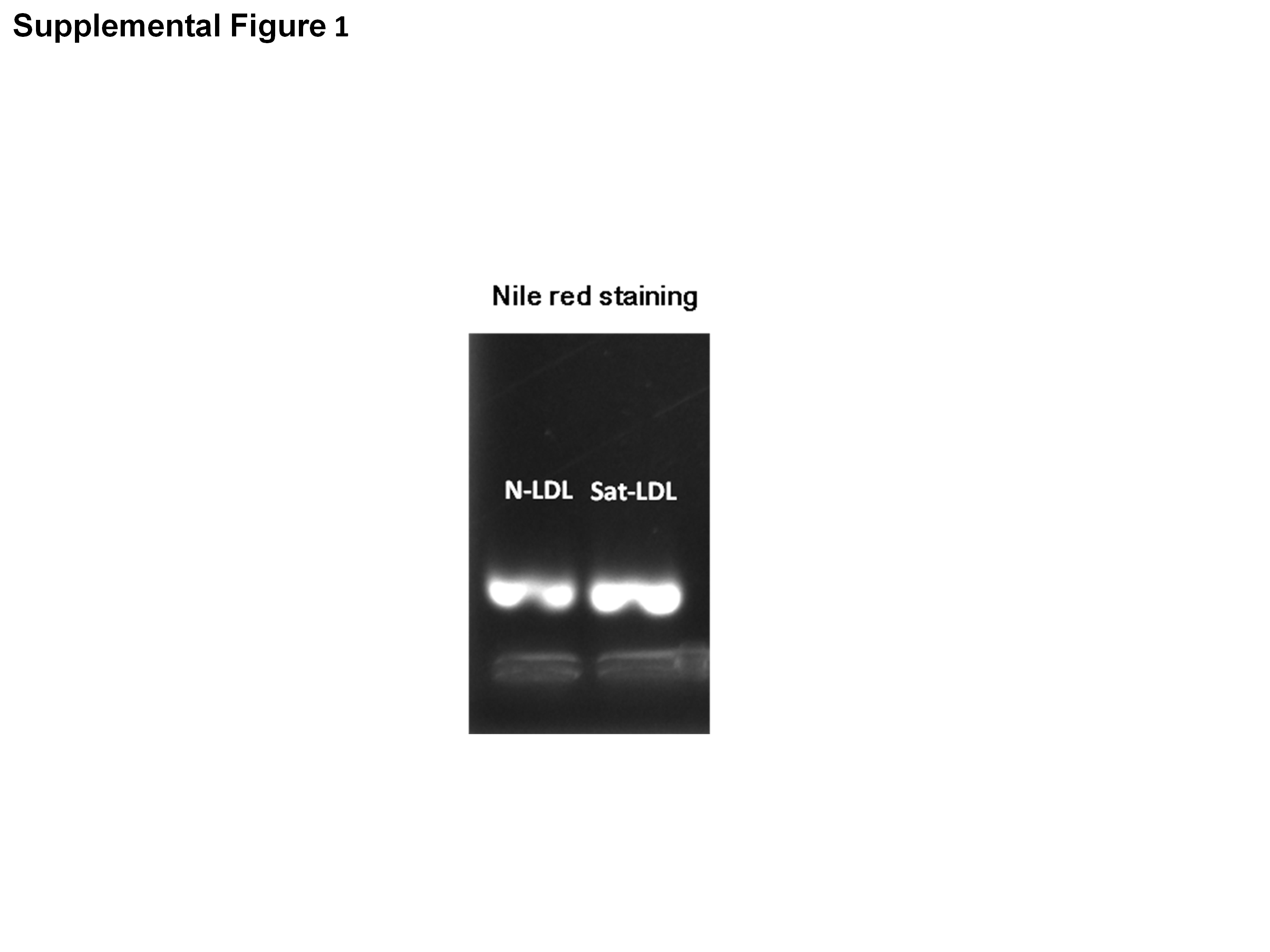


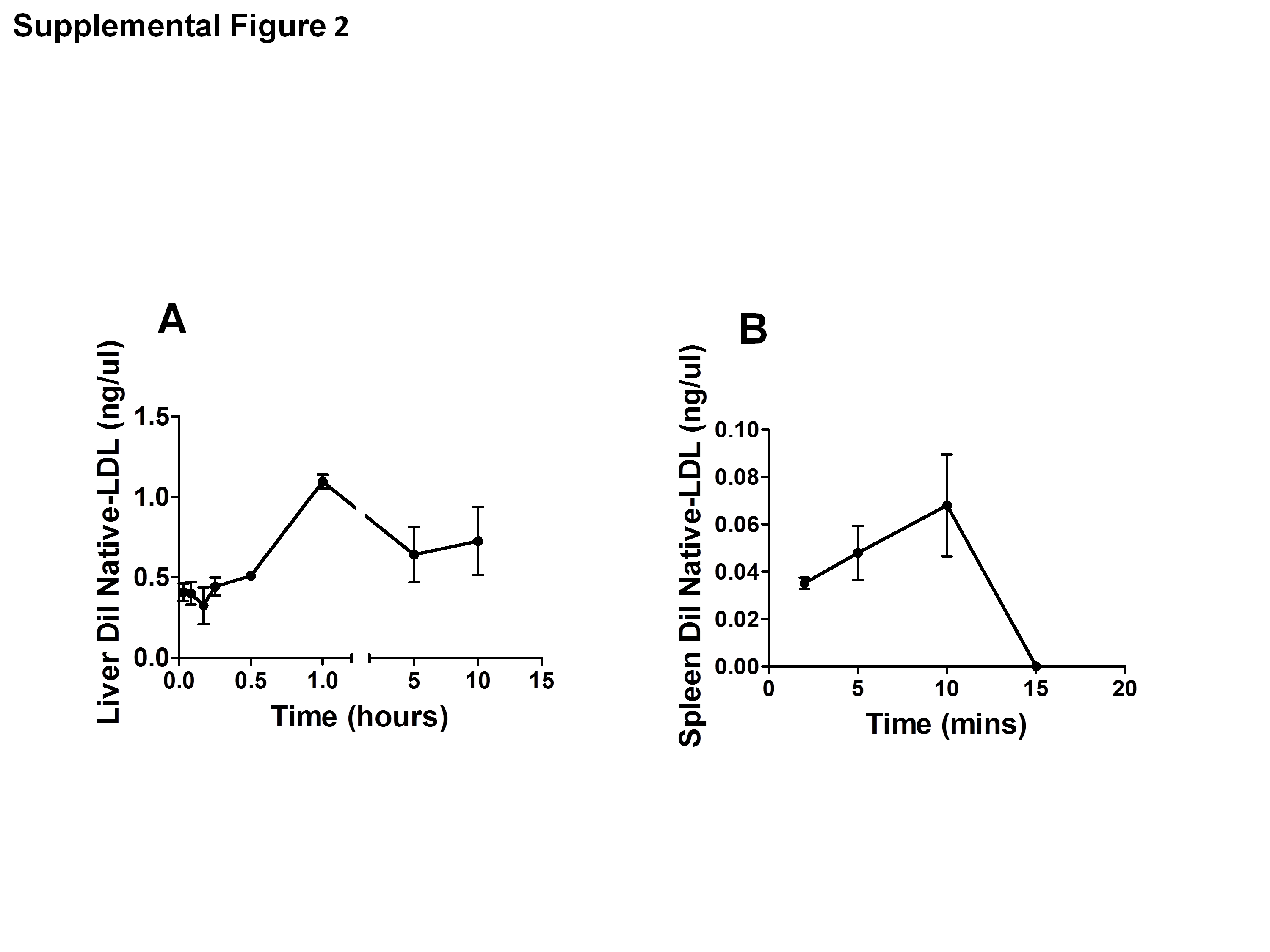


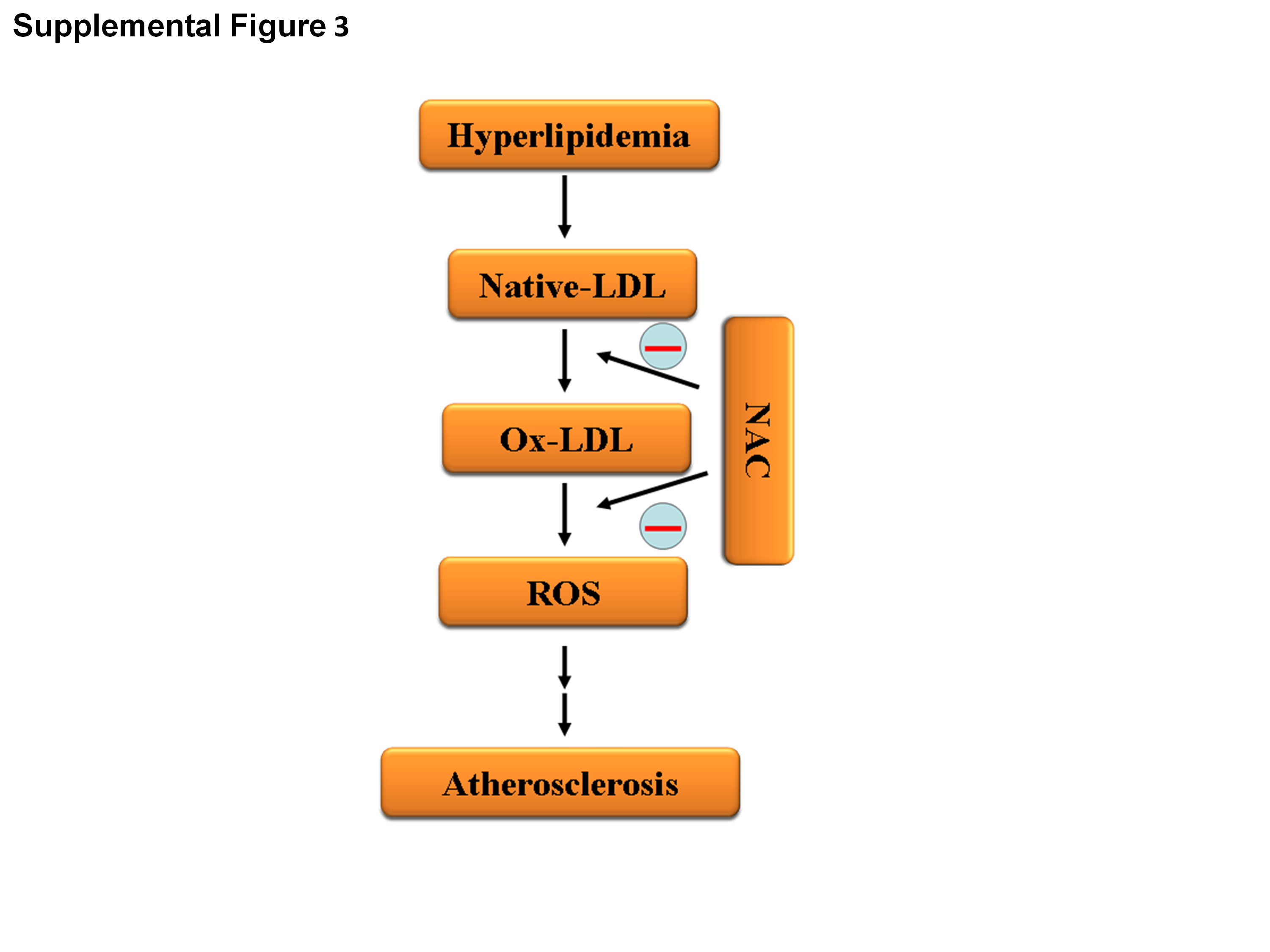

Supplement: Supplementary Dataset [file srep16339-s1.doc]
